# Visual tuning and metrical perception of realistic point-light dance movements

**DOI:** 10.1038/srep22774

**Published:** 2016-03-07

**Authors:** Yi-Huang Su

**Affiliations:** 1 Department of Movement Science, Faculty of Sport and Health Sciences, Technical University of Munich, Munich, Germany

## Abstract

Humans move to music spontaneously, and this sensorimotor coupling underlies musical rhythm perception. The present research proposed that, based on common action representation, different metrical levels as in auditory rhythms could emerge *visually* when observing structured dance movements. Participants watched a point-light figure performing basic steps of Swing dance cyclically in different tempi, whereby the trunk bounced vertically at every beat and the limbs moved laterally at every second beat, yielding two possible metrical periodicities. In Experiment 1, participants freely identified a tempo of the movement and tapped along. While some observers only tuned to the bounce and some only to the limbs, the majority tuned to one level or the other depending on the movement tempo, which was also associated with individuals’ preferred tempo. In Experiment 2, participants reproduced the tempo of leg movements by four regular taps, and showed a slower perceived leg tempo with than without the trunk bouncing simultaneously in the stimuli. This mirrors previous findings of an auditory ‘subdivision effect’, suggesting the leg movements were perceived as beat while the bounce as subdivisions. Together these results support visual metrical perception of dance movements, which may employ similar action-based mechanisms to those underpinning auditory rhythm perception.

Musical rhythms are composed of hierarchical temporal modules such as pulse, beat, and meter^1^,^2^. These organized temporal patterns constitute an important factor in music that makes us move^3^: As observed in everyday life, listening to music induces spontaneous body movements that are often periodic and coordinated with the musical beat^4^,^5^. When adults move to music, different metrical levels of the rhythm can be simultaneously reflected in different kinds of body movements^6^. Notably, musical rhythms couple not only humans’ external movements, but also the internal motor system. Mounting research has shown that, in the absence of overt movements, cortical and subcortical motor areas are engaged by the presence of an auditory beat^7^, and the activations are modulated by metrical complexity of auditory rhythms^8^,^9^. Similarly, cortical oscillations^10^ and evoked potentials^11^ are found to reflect tuning to different metrical levels of the rhythm. Adding to the evidence of sensorimotor coupling are findings that explicitly moving to the beat also modulates perception of pulse, meter, and timing of auditory rhythms^12^,^13^,^14^, suggesting the bidirectional nature of this interaction. Together these observations support the notion that music perception, especially regarding its rhythms, represents a manifestation of embodied cognition^3^.

The idea that human movements embody the structure of musical rhythms can be accommodated in a broader framework of common coding for action and perception^15^,^16^,^17^. That is, a strong link exists between how rhythms are perceived and how humans (intend to) move to rhythms, as these processes share a common representation in the sensorimotor system^18^,^19^. This proposal connects in part with the action recognition and simulation literature that, originating from the visuo-motor domain, shows overlapping internal motor representation of actions by execution and by observation^20^,^21^. This finding has also been extended to the auditory modality^22^,^23^ and, indeed, multisensory (audiovisual) scenarios^24^,^25^. Namely, action representation can be triggered by one or more sources of sensory information associated with the action.

Although an action can be represented in various sensory modalities, the temporal characteristics of human movement – in particular its rhythm – has mainly been linked to auditory perception in a musical context. For example, humans prefer a range of beat tempo around 1.5–2.5 Hz (inter-beat intervals between 400 ms and 700 ms) in musical rhythms^26^,^27^, typically measured by finger tapping tasks^28^,^29^, which corresponds roughly to the preferred frequency of locomotion (~2Hz)^30^. Recent research has demonstrated promising visual rhythm perception and synchronization with a periodically moving object, such as a bouncing ball^31^,^32^, and that humans can visually entrain to a simple periodicity of another individual’s movement, such as finger-tapping trajectory, leg oscillation, or body sway (see section 3.2 of^28^). Despite these findings, the extent to which this process applies to the rhythmicity of more complex human movements is unknown. One critical question, then, is whether human movements as visual information can communicate metrical structure encompassing more than one periodicity, similar to that in musical rhythms, and whether observers perceive such visual rhythms using similar mechanisms as those for the auditory counterpart.

The present research addressed this issue by integrating frameworks of embodied musical rhythm and the action-perception coupling. The general hypothesis was formed as following: The internal motor representation of musical rhythms should be elicited not only auditorily by listening to rhythms to which one would move, but also *visually* by observing movements corresponding to how one would move along the musical rhythms. As such, it was expected that humans can perceive visual rhythms by observing music-induced movements. More importantly, this process may parallel auditory rhythm perception, such that different metrical modules can be visually extracted.

Dance movements serve suitable visual stimuli to test this hypothesis, as they consist of structured movement patterns performed in time to music and are – albeit to various degrees depending on the repertoire – temporally coordinated with musical rhythms^33^,^34^. In particular, dance movements are expected to communicate visual rhythms if they correspond to how humans would naturally move to music. Given previous findings that vertical trunk movements tend to be synchronized to a regular pulse^6^,^35^, while lateral movement patterns of the limbs can correspond to higher metrical grouping^6^,^34^, two kinds of dance movements encompassing these components were selected as visual stimuli for the present study: *Charleston*, and *Balboa* (Fig. 1). Both dances belong to the class *swing dance*, typically accompanied by swing music of a 4/4 musical meter^36^. A sequence of basic steps corresponds temporally to eight beats. The basic steps of each dance consist of two movement components simultaneously: (1) a regular, vertical bounce carried out by repetitive knee flexion and extension^37^, which moves the trunk down and up accordingly (where the bounce is most evident, Fig. 2c), and (2) regular lateral movements of the limbs (Fig. 2a,b). Critically, the trunk bounces at every beat, whereas the limbs move at every second beat. As a result, two parallel, hierarchical metrical levels may be visually extracted in each dance: the periodicity of the bounce, and the periodicity of the limb movement, with the latter being twice as slow as the former.

Three specific hypotheses were made regarding the proposed visual rhythm perception in these dance movements: (1) The bounce of the trunk should communicate a regular pulse^35^, whereas the limb movement pattern should communicate metrical accents (at every second pulse) that may serve as beat. (2) In terms of the beat, which was assumed to be most salient in the leg movement based on our recent finding^38^, the patterning may be composed in two ways. In one, the legs or arms stretch in a torso-centered manner in the horizontal plane with relatively large trajectories (e.g., periodic arm swinging or leg kicking), as found in *Charleston* (Figs 1a and 2a). In the other, the pattern is mainly derived from the footstep-like movements (similar to locomotion), in which the feet occupy different locations on the ground in a timed manner, as found in *Balboa* (Figs 1b and 2b). (3) It was further hypothesized that translational motion (TM) in the horizontal plane (Fig. 2a,b) should be more important for *Balboa* in communicating a beat, as the periodicity of leg movements likely arises from the spatial positions on the ground (e.g., successive footsteps forming a square). By contrast, the periodicity may already be evident in the limb trajectories of *Charleston* whether or not the whole body moves horizontally.

Two different tasks were conducted to test these hypotheses. Experiment 1 started by investigating whether observers could visually tune to different metrical periodicities in each dance movement, whether they preferred one tempo (e.g., that of the limbs) over the other (e.g., that of the trunk) in the same movement, and whether their chosen metrical level varied depending on the global movement tempo. The task could be seen as the visual analogue of the ‘beat finding task’ often adopted to probe auditory tuning in music^13^,^39^,^40^. Participants observed long sequences of point-light *Charleston* or *Balboa* movement repeated cyclically in six different tempi, with or without TM, and were asked to tap to a tempo in the movement that felt natural to synchronize with. If visual tuning resembles its auditory counterpart, then observers should be able to extract both metrical levels (i.e., the trunk and the leg tempi) visually^29^. Of interest, then, was the percentage of tuning to either metrical level as a function of the movement tempo and TM. The following was predicted for this task: If a range of optimal tempo existed in visual rhythms as in auditory ones^27^, observers may switch from one level to the other when only one of them fell in this range. In addition, the choice of tuning may be related to each individual’s preferred tempo.

Experiment 2 also applied the logic of an established auditory paradigm to examine whether the two visual metrical levels were perceived simultaneously as beat (leg movement) and subdivisions (trunk movement). According to previous findings of the auditory ‘subdivision effect’^41^,^42^, the same beat tempo is perceived as slower with than without subdivisions between successive beats. Here, observers watched a short sequence of the dance movement that was either as naturally performed, or manipulated to eliminate the bounce of the trunk, after which they were asked to reproduce the tempo of the leg movement by making four finger taps. The perceived tempo of the leg movements, indexed by the reproduced tempo, was hypothesized to be slower in the former condition.

## Results

### Experiment 1: Tuning to the dance movements

Based on tuning percentages from successful trials, three groups of tuning behaviors were identified amongst the thirty participants:

Group 1: those who could tune to either the bounce or the limb movement depending on the tempo of the movement sequence (*N* = 16).

Group 2: those who only tuned to the limb movement (*N* = 8), i.e., zero percentage of tuning to the bounce across all the conditions.

Group 3: those who only tuned to the bounce (*N* = 6), i.e., zero percentage of tuning to the limbs across all the conditions.

#### Tuning behavior of Group 1

For Group 1, the individual percentages of tuning were submitted to a 2 (Dance style) × 2 (TM) × 6 (Tempo) × 2 (Tuning level: bounce or limbs) repeated-measures ANOVA. Greenhouse-Geisser correction was applied to the *p* values of effects involving Tempo (also for all the other ANOVAs reported in this study). A main effect of Tuning level was found, *F*(1, 15) = 5.14, *p* = 0.039, *η*_*p*_^*2*^ = 0.26, showing overall a greater percentage of tuning to the limb (*M* = 64.7%) than to the bounce tempo (*M* = 29.7%). The three-way interaction Dance style × Tempo × Tuning level was significant, *F*(5, 75) = 3.38, *p* = 0.025, *η*_*p*_^*2*^ = 0.18, as well as the two-way interaction Tempo × Tuning level, *F*(5, 75) = 4.89, *p* = 0.025, *η*_*p*_^*2*^ = 0.25. There was also a significant TM × Tuning level interaction, *F*(1, 15) = 4.83, *p* = 0.04, *η*_*p*_^*2*^ = 0.24 (Fig. 3).

Following the three-way interaction, the 6 (Tempo) × 2 (Tuning level) repeated-measures ANOVAs were conducted for each Dance style separately. A significant interaction between Tempo and Tuning level was only found for Balboa, *F*(5, 75) = 6.73, *p* = 0.009, *η*_*p*_^*2*^ = 0.31, but not for Charleston, *F*(5, 75) = 1.80, *p* = 0.12, *η*_*p*_^*2*^ = 0.11 (Fig. 3). Following this two-way interaction in Balboa, two separate one-way ANOVAs were conducted for each Tuning level, showing that while Tempo had no effect on tuning percentages to the limbs, *F*(5, 75) = 2.37, *p* = 0.13, *η*_*p*_^*2*^ = 0.14, Tempo had an effect on tuning percentages to the bounce, *F*(5, 75) = 10.76, *p* = 0.001, *η*_*p*_^*2*^ = 0.42. Post-hoc pairwise comparisons (with Bonferroni corrections) revealed differences of tuning percentages between the fastest tempo (IBI = 300 ms) and the slowest tempo (IBI = 550 ms), *p* = 0.028, as well as between the second fastest (IBI = 350 ms) and the two slowest tempi (IBI = 500 ms and 550 ms), *p* = 0.046 and *p* = 0.021, respectively. There was also a trend of difference between the fastest tempo (IBI = 300 ms) and the second slowest (IBI = 500 ms), *p* = 0.074, and a trend of difference between IBI = 450 ms and IBI = 550 ms, *p* = 0.06.

Finally, following the significant interaction between TM and Tuning level, a post-hoc one-way ANOVA was conducted for each Tuning level separately. For tuning to the bounce, there was more tuning with than without TM, *F*(1, 15) = 6.40, *p* = 0.02, *η*_*p*_^*2*^ = 0.30, whereas the effect of TM did not reach significance for tuning to the limbs, *F*(1, 15) = 3.15, *p* = 0.096, *η*_*p*_^*2*^ = 0.17 (Fig. 4).

#### Correlation between tuning pattern and SMT in Group 1

To examine whether the choice of metrical level correlated with each participant’s preferred tempo as measured by the spontaneous motor tempo (SMT) task, correlational analyses were conducted at an individual level (*N* = 16) between the following two measurements: (1) the SMT as indexed by the mean ITI (in ms), and (2) the difference between the percentage of tuning to the limbs and to the bounce (by subtracting the latter from the former). A positive correlation would suggest that a slower preferred tempo (i.e., longer ITI) was correlated with a higher tendency of tuning to the limbs compared to the bounce. Correlational analyses were computed for each of the 2 (Dance style) × 2 (TM) × 6 (Tempo) conditions separately. Table 1 summarizes results of the analyses, showing in general more positive correlations at slower movement tempi (original *p* < 0.05, and *p* = 0.0605 following FDR adjustment).

In sum, for participants who could tune to both metrical levels, they more often tuned to the limb movement than to the bounce, which was true for both dance styles across all movement tempi. Especially for *Balboa*, the frequency to tune to the bounce increased as the movement became slower. In addition, for both dance styles there was more tuning to the bounce with than without TM. Finally, the tendency to switch between the two metrical levels was related to each individual’s preferred tempo, such that more tuning to the limbs compared to the bounce appeared correlated with a slower SMT. This correlation seemed more evident when the dance movement was slower.

#### Tuning behavior of Group 2

For those who only tuned to the limb movement, the individual percentages of tuning were submitted to a 2 (Dance style) × 2 (TM) × 6 (Tempo) repeated-measures ANOVA. A significant Dance style × Tempo interaction was found, *F*(5, 35) = 4.45, *p* = 0.03, *η*_*p*_^*2*^ = 0.39. Two follow-up one-way ANOVAs for each Dance style separately revealed that while there was no effect of Tempo on tuning percentage in Charleston, *p* = 0.37, the effect of Tempo was significant in Balboa, *F*(5, 35) = 4.81, *p* = 0.02, *η*_*p*_^*2*^ = 0.41. Post-hoc comparisons (Tukey HSD) showed a lower tuning percentage to the fastest movement tempo (IBI = 300 ms) than to the two slower movement tempi (IBI = 500 and 550 ms), in both cases *p* = 0.04. As there was no switching between different metrical levels in this group, the lower percentage of tuning at the fastest movement tempo rather resulted from less successful tuning in this condition (Fig. 5a).

#### Tuning behavior of Group 3

For those who only tuned to the bounce, the 2 (Dance style) × 2 (TM) × 6 (Tempo) repeated-measures ANOVA did not reveal any significant effect of the variables (Fig. 5b).

#### Comparison of SMT amongst the three groups

Finally, to examine whether tuning behaviors were associated with individuals’ preferred tempo, mean SMTs were compared between pairs of the three tuning groups (two-sample t-tests, two-tailed, Bonferroni corrected *p* values). It was found that Group 2 (limb tuning) had a slower SMT (mean ITI = 688 ms) than either Group 1 (trunk tuning, mean ITI = 456 ms), *t*(12) = 4.53, *p* = 0.001, or Group 3 (tuning to both, mean ITI = 493 ms), *t*(22) = 4.31, *p* < 0.001, while Group 1 and Group 3 did not differ from each other statistically. Thus, those who only tuned to the limb movements appeared to have a slower intrinsically preferred tempo than the others.

### Experiment 2: Metrical tempo perception in dance movements

The individual means of reproduced tempo, as indexed by percentages of deviation from perfect reproduction, was submitted to a 2 (Dance style) × 2 (Trunk movement) × 6 (Tempo) repeated-measures ANOVA. The main effect of Trunk movement was significant, *F*(1, 19) = 7.14, *p* = 0.015, *η*_*p*_^*2*^ = 0.27, showing a slower reproduced leg tempo (i.e., longer mean ITI) for conditions with trunk movement than without.

A significant three-way interaction was also found, *F*(5, 95) = 2.88, *p* = 0.036, *η*_*p*_^*2*^ = 0.13. Following that, a 2 (Trunk movement) × 6 (Tempo) ANOVA was conducted for each Dance style separately: For Charleston, there was a significant Trunk movement × Tempo interaction, *F*(5, 95) = 3.23, *p* = 0.03, *η*_*p*_^*2*^ = 0.15, as well as a significant main effect of Trunk movement, *F*(1, 19) = 12.14, *p* = 0.002, *η*_*p*_^*2*^ = 0.39 (Fig. 6a). For Balboa, though, neither effect was significant, *p* > 0.1 and *p* > 0.3, respectively (Fig. 6b). For Charleston, further post-hoc one-way ANOVAs conducted for each tempo revealed that the reproduced tempo was slower with than without trunk movement specifically for the following tempi: IBI = 450 (*p* = 0.018), IBI = 400 (*p* < 0.001), and marginally so at IBI = 350 (*p* = 0.054).

In short, while the reproduced tempo of the leg movement was generally slower in conditions with concurrent trunk movement than without, this effect was most prominent for Charleston dance at a range of moderate tempi.

## Discussion

Two experiments examined the hypothesis that, when watching dance movements composed of kinematic patterns resembling how humans spontaneously move to music (e.g., vertical movements being coordinated with the pulse while lateral movements with higher-level metrical accent)^4^,^6^, observers can perceive the rhythm of dance movements by means of action-perception coupling^15^,^17^. Accordingly, observers may extract different metrical periodicities in these movements in a similar manner as hearing the rhythm in music. In both experiments, participants watched a PLF performing repetitive cycles of basic steps from *Charleston* and *Balboa* dance in six different tempi. In Experiment 1, participants freely tuned to a tempo in each movement, and three different tuning behaviors were identified: those that always tuned to the bounce (the fastest pulse level), those that always tuned to the lateral limb movement (the slower, higher metrical level), and those that tuned to one level or the other depending on the movement tempo (about twice as many as each other group). Experiment 2 further complemented the finding of two visual metrical levels by showing that the tempo of the same leg movements was perceived to be slower with than without the trunk bouncing simultaneously. This pattern is consistent with the subdivision effect found in auditory rhythm perception^41^, suggesting that the leg movements marked the beat while the trunk movements were perceived as the subdivisions between successive beats.

The three different visual tuning behaviors found in Experiment 1 well mirror previous results of auditory beat-finding in music^39^,^40^. While considerable individual differences exist in those auditory studies as well as in the present visual one, Martens^40^ reported the same three groups of tuning strategies in music. This serves the first indication that visual rhythm perception in certain dance movements employ similar mechanisms as their auditory counterpart in music, where multiple levels of periodicity can be extracted. The choice may depend on each individual’s preferred tempo^27^,^43^, age^44^, musical training^29^, the nominal tempo^39^ as well as rhythmic complexity or accentuation^13^,^45^ of the stimuli, amongst other factors. Whereas Martens^40^ failed to find association between the chosen tempo and individual’s SMT, the present study does demonstrate such a link in two findings: First, those who only tuned to the limbs (higher metrical level) had on average a slower SMT than the others. Secondly, amongst those who tuned to both levels, the tendency to switch to the limbs when the movement tempo was slow also appeared to correlate with a slower SMT. This latter finding may be explained as following: When the movement was fast, it was equally likely for observers to choose the limb tempo, as the bounce was faster than would be comfortable to synchronize with^26^. When the movement was slow, however, the bounce tempo became more salient while the limb tempo might appear slower than optimal. In this case, those who had a slower intrinsic tempo might still be more driven to the limb tempo rather than the bounce.

Amongst those who tuned to both metrical levels, their pattern also draws interesting parallels to what has been found in auditory rhythms. First, observers tuned more often to the limbs than to the bounce, which may have resulted from the limb (especially leg) movements being perceived as the more salient beat. This fits well with our previous result that observers oriented to regular leg movements (and *not* the arms), if present, as temporal reference to assist visual timing of a short dance sequence^38^. As shown in earlier auditory findings, when a particular metrical level is accentuated by nature of its physical properties^26^,^39^, it may draw listeners to that level even if it does not fall within the range of optimal tempo for synchronization^27^. This may explain why the leg movements were perceptually prioritized as the visual beat regardless of their tempo. Next, especially in *Balboa*, the tendency to tune to the bounce increased as the movement tempo decreased. This result suggests that, as the movement becomes slower (e.g., at the inter-bounce interval of 500 and 550 ms), the limb tempo (inter-limb interval of 1000 and 1100 ms) may appear too slow for comfortable synchronization, while the faster level (the bounce) may seem more natural. The frequency to tune to the latter thus increased. As such, it would seem as if there is also a range of optimal tempo for perceptual and motor synchronization in visual dance rhythms, which may partially overlap with that found in music^27^. Notably, this effect was most evident in *Balboa*, suggesting that it depended on the saliency of the leg movement as visual beat. When the leg movements consisted of large trajectories as in *Charleston*, the bounce could not seem to compete with them perceptually; when the amplitude of the leg movements was smaller as in *Balboa*, the bounce in proper tempi started to attract more attention to emerge as beat^46^.

The subdivision effect revealed in Experiment 2 further confirms visual metrical hierarchy in dance observation. Observers implicitly incorporated the timing of the concurrent trunk bounce in their perception of leg tempo, which served to subdivide successive leg movements. Consequently, the leg tempo was perceived to be slower than when the bounce was removed. As all the kinematic parameters of the legs remained identical across the trunk manipulations, the effect was specific to the presence or absence of the bounce. Interestingly, this effect was stronger in *Charleston* when the inter-limb intervals fell between 700 ms and 900 ms, suggesting that this might be the range of tempo within which the two metrical levels can be most optimally perceived in parallel. When the movement was faster than this range, the bounce might be too fast or – due to biomechanical constraint in the performance – of too little amplitude to be salient enough in perception. It is less clear why movements slower than this range did not yield evident subdivision effect, as the bounce should have been most obvious in the slow tempi. One could speculate that, when the legs moved slowly, it was possible to subdivide the leg trajectory by means of its kinematic cues (e.g., middle position or minimal velocity^35^) in the absence of the bounce. As to why the effect was less evident in *Balboa*, one possible explanation is that, as the legs moved in steps, the bounce information was somewhat preserved in the knee movements, which made up partially for the absence of the trunk pattern.

Finally, contrary to the hypothesis, TM did not interact with dance style, suggesting that the same mechanism was applied to extract periodicity for both leg movement types. The process seemed to rely on the entire leg trajectories rather than the end spatial positions, also for footstep-like movements as in *Balboa*. Unexpectedly, TM contributed to more tuning to the bounce compared to the absence thereof. This can be interpreted as the spatial frame of the whole body moving forward and backward adding metrical accents to the bounce, making it more salient and beat-inducing. Thus, as opposed to the hypothesized effect on beat perception of leg movements, the horizontal spatial information of TM assisted in highlighting the periodicity of the vertical bounce instead.

The present research demonstrates that dance observation provides an ecologically plausible scenario, in which mechanisms of rhythm perception apply to the visual modality in a comparable manner to those for music. Overall the results support the proposal that, as with auditory musical rhythms, visual rhythms derived from dance movements can present multiple metrical periodicities unfolding in parallel over time. In movements with repetitive and structured patterns, one metrical level can emerge visually as beat while another as underlying subdivisions. This may indeed be because dance movements arise as a response to – and are performed to express – musical structures^33^,^34^, which consist of such temporal hierarchy. In light of action-perception coupling and embodied musical rhythms^17^, observing dance that contains patterns resembling humans’ spontaneous movements to music^6^ likely simulates these movements accordingly in the internal motor system^20^,^47^. As these movements are temporally associated with musical rhythms, it may be further postulated that such visual input shares overlapping motor representations with auditory musical rhythms^7^,^25^,^48^. In the context of music and dance, rhythm perception can be seen as multisensory based on convergent (internal or external) motor correspondence. Various inter-modal and sensorimotor interactions are worth further investigations: For example, given the present results, follow-up research has been planned to examine whether sensorimotor synchronization to visual dance stimuli would entail similar mechanisms as those for auditory rhythms. Also informative would be to what extent visual rhythm processing of dance stimuli is modulated by dance and music expertise respectively, as it may tap onto integrated skills required for both^49^.

## Method

### Experiment 1: Tuning to the dance movements

#### Participants

Thirty young, healthy volunteers (nine male, mean age 24.5 years, SD = 4) took part in this experiment. All participants were naïve of the purpose, gave written informed consent prior to the experiment, and received an honorarium of 8 € per hour for their participation. Participants were not pre-screened for musical or dance training, which ranged from zero to twenty years (all amateurs). The mean duration of music and dance training was 7.6 years (SD = 5.7) and 2.8 years (SD = 5.4), respectively. There were more musicians (25 out of 30) than dancers (11 out of 30), amongst whom eight had learned both. The study had been approved by the ethic commission of Technical University of Munich, and was conducted in accordance with the ethical standards of the 1964 Declaration of Helsinki.

#### Stimuli and materials

The visual stimuli consisted of a human point-light figure (PLF) performing two kinds of dance movements derived from the basic steps of *Charleston* and *Balboa* repertoire. For each dance, one cycle of the movement sequence corresponds temporally to 8 metronome beats.

**Figure 1:**
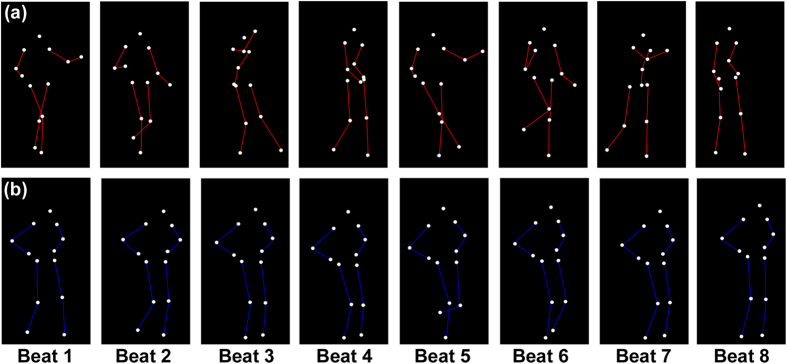
Illustration of the point-light motion stimuli used in both experiments, showing one cycle of (**a**) *Charleston* dance and (**b**) *Balboa* dance, respectively. Each cycle corresponds to eight metronome beats. The eight columns for each dance are taken from the frames in the stimuli representing the posture at each beat. In both examples, the figure moves with translational motion (TM) horizontally. The sequences illustrate the limb movement patterns, while the regular bounce of the trunk is not immediately obvious here. The lines connecting the joints (red for *Charleston* and blue for *Balboa*) are drawn here for the purpose of visualization, which did not exist in the visual stimuli as displayed in the experiments.

**Figure 2:**
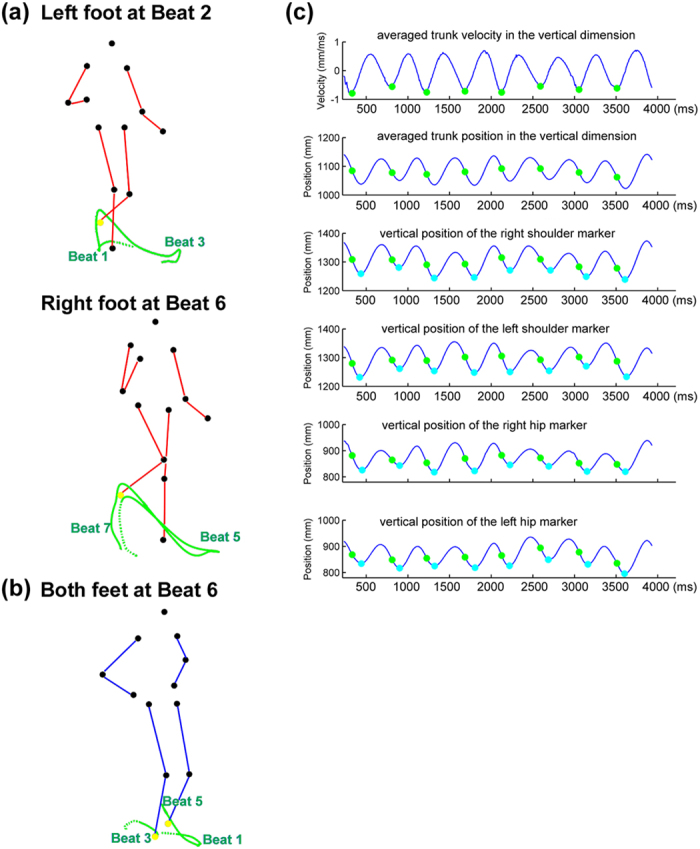
The trajectory and kinematics of the point-light motion stimuli, with inverted colors of the background and the markers for easier visualization: (**a**) The tracked trajectories, shown in green lines, of the left foot (upper panel) and the right foot (lower panel) in *Charleston*, plotted on the frame at Beat 2 and Beat 6, respectively. The left foot positions at Beat 1 and 3, and the right foot positions at Beat 5 and 7, are noted relative to each entire trajectory. The tracked foot marker is shown in yellow in the respective panel. The dotted trace represents the trajectory leading up to the earliest beat in each panel. The two frames are taken from the same perspective relative to the PLF movement, and translational motion (TM) can be seen here as the PLF has moved forward in the lower panel relative to the upper one; without TM there is no obvious horizontal displacement as such. (**b**) The tracked trajectories (green lines) of the right and left foot (both in yellow) in *Balboa*, plotted on the frame at Beat 6. TM can also be seen here as the horizontal displacement of both feet relative to the starting position (the beginning of each dotted trace). (**c**) The kinematic profile of the trunk from one cycle of *Charleston* motion stimuli (with TM, IBI = 450 ms), showing 8 bounces in the vertical dimension. The first and second panels show the vertical velocity and position, respectively, of the four trunk markers averaged against the time vector (X-axis), whereas the four subsequent panels show the vertical positions of each of the four markers separately. The green and blue circles mark the point of peak velocity and the point of lowest position in each bounce, respectively. The trunk kinematic pattern in *Balboa* is the same.

**Figure 3:**
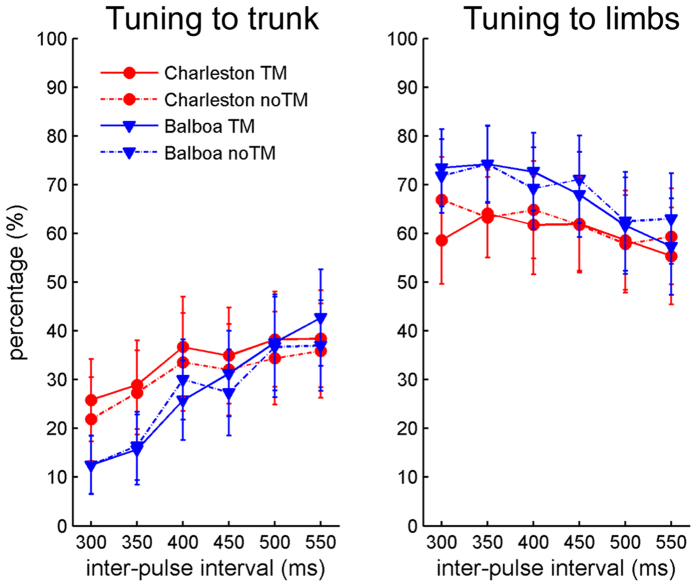
Results of Experiment 1: Mean percentages of tuning to the trunk and tuning to the limbs in the same participants (Group 1, *N* = 16) as a function of the visual stimulus tempo, for each stimulus condition separately. The stimulus tempo is indexed nominally by the inter-pulse interval of the metronome to which the PLF moved. The X-axis from left to right thus denotes the visual stimulus tempo from fast to slow. Error bars represent standard error of the means.

**Figure 4:**
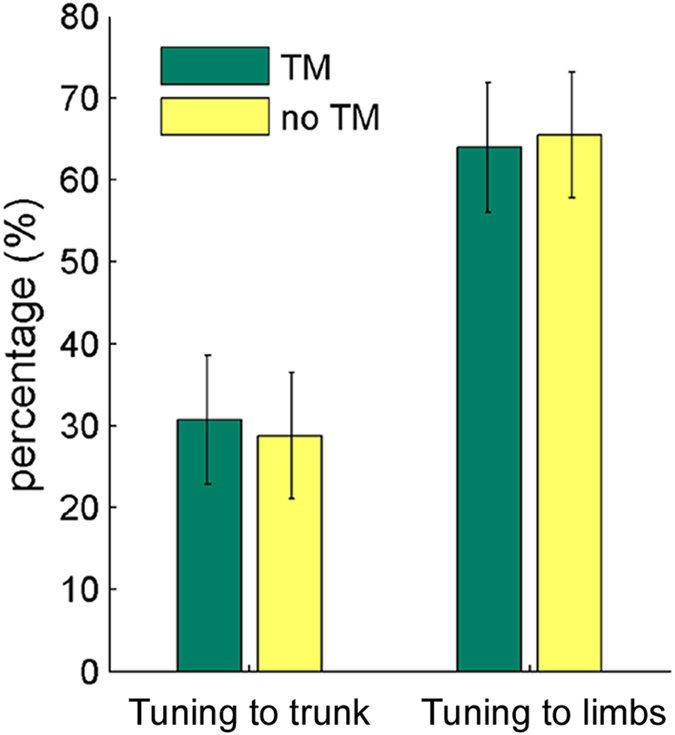
Results of Experiment 1: Mean percentages of tuning to the trunk and tuning to the limbs in movements with or without TM, for Group 1 (*N* = 16). Error bars represent standard error of the means.

**Figure 5:**
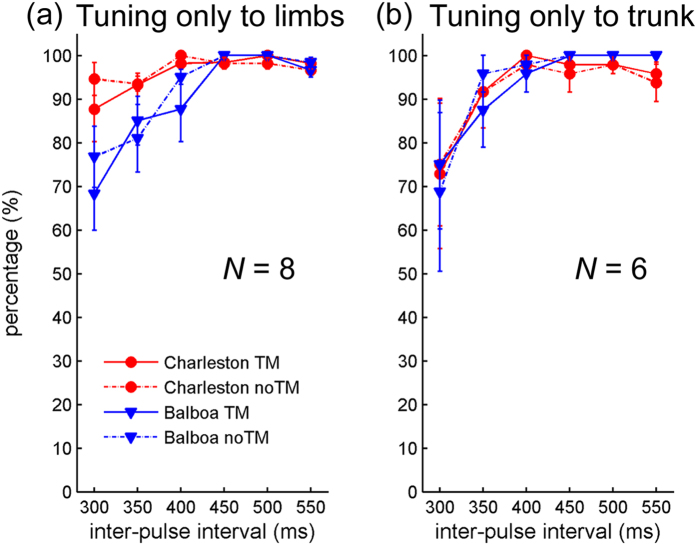
Results of Experiment 1: Mean percentages of tuning for (**a**) those who only tuned to the limbs (Group 2, *N* = 8) and (**b**) those who only tuned to the trunk (Group 3, *N* = 6), as a function of visual stimulus tempo. Error bars represent standard error of the means.

**Figure 6:**
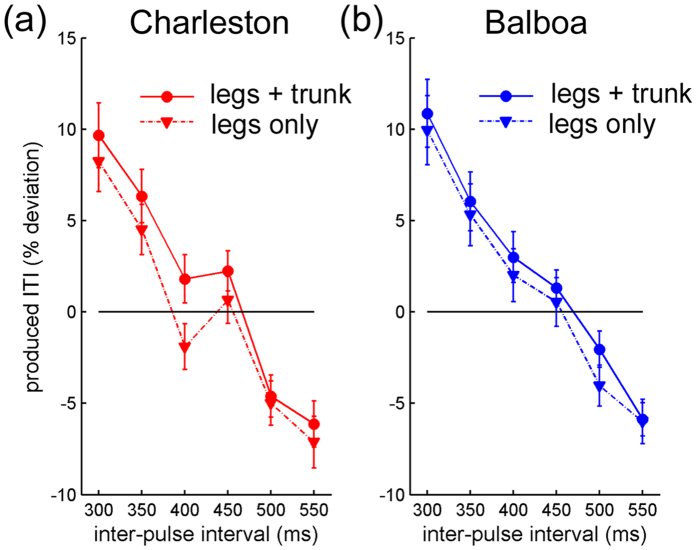
Results of Experiment 2: Mean reproduced tempi of the leg movement in conditions with or without the trunk movement, as a function of the visual stimulus tempo, for (**a**) *Charleston* dance and (**b**) *Balboa* dance. The reproduced tempo is indexed on the Y-axis as the deviation (in %) of the mean reproduced ITI from the nominal stimulus IBI. The horizontal black line represents perfect tempo reproduction.

The *Charleston* (Figs 1a and 2a) is characterized first by a regular body bounce in the vertical dimension in time with every beat, which is most evident in the trunk movement (Fig. 2c). In addition, the arms and the legs move laterally, whose patterns can be grouped in four at a tempo that is twice as slow as the bounce, i.e., one leg or arm movement every two bounces. The leg movement consists of the left leg stretching backward for a kick (Beat 1), stretching forward for another kick (Beat 3), followed by the right leg doing similar patterns first forward (Beat 5) and then backward (Beat 7). The arm movement consists of the two arms swinging in opposite directions simultaneously, with the left arm swinging forward (Beat 1 and Beat 5) and backward (Beat 3 and Beat 7) and the right arm swinging backward (Beat 1 and Beat 5) and forward (Beat 3 and Beat 7).

The *Balboa* (Figs 1b and 2b) consists mainly of trunk and leg movements. For the present study, the arms were choreographed to remain still with the palms placed upon the hips throughout the sequence. As in *Charleston*, in *Balboa* the trunk also bounces regularly at every beat. Meanwhile, the legs carry out footstep-like movements originally at every beat. For the purpose of presenting two temporal levels comparable to those in *Charleston*, the *Balboa* here was modified such that there was one footstep every two bounces. The leg movements are thus as following: left foot stepping forward (Beat 1), right foot stepping forward (Beat 3), left foot sliding backward (Beat 5), left foot sliding forward to reach the ground (Beat 7). In the next 8-beat cycle, the leg pattern is carried out in a mirrored manner by the opposite feet: right foot stepping backward (Beat 1), left foot stepping backward (Beat 3), right foot sliding forward (Beat 5), and right foot sliding backward to reach the ground (Beat 7).

The stimuli were constructed by recording a swing dancer (amateur with 5 years of regular training) performing the choreographed movements paced by metronomes of six different tempi, corresponding to an inter-beat interval (IBI) of 300, 350, 400, 450, 500, and 550 ms, respectively. Two movement variations were included for each dance, such that the same sequence was performed either with the whole body moving forward and backward along the sagittal plane as the feet moved along two imaginary parallel lines on the ground, one for each foot (with TM, see also Fig. 2a,b), or with the body remaining mostly in place (without TM). The dancer had practiced all the movement variations in all tempi prior to the recording. The movements were recorded by a 3D motion capture system (Qualisys Oqus, 8 cameras) with a sampling rate of 200 Hz. In total 13 markers were attached to the head, shoulders, elbows, wrists, hips, knees, and feet during the recording^50^. For each movement variation at each tempo, the dancer was recorded performing the sequence repetitively and continuously for at least 60s, yielding 10 to 24 cycles for each recorded movement sequence.

One “best movement cycle” was selected from each recording to be the visual stimulus, based on criteria of several kinematic parameters of the motion-captured data: (1) The duration of one complete cycle and its deviation from eight times the respective metronome IBI, (2) the mean temporal interval and variability of the eight successive bounces, as calculated by both the end position and the peak velocity cues^35^ of the four trunk markers (shoulders and hips), (3) the mean temporal interval and variability of the four successive leg movements, calculated by both end position and peak velocity cues of the foot markers, and (4) for *Charleston*, the mean interval and variability of the successive arm movements calculated from the hand markers. An integrated index was subsequently computed, in which equal weight was given to the duration deviation and the variability of all these parameters. For each movement variation at each tempo, the cycle that received the lowest index was selected as the best cycle. Each best cycle was presented as visual stimuli without further editing, except for slight spatial interpolations to ensure the movement can be looped smoothly when presented in continuous cycles (see also^35^). Thus, as opposed to previous work that imposed additional spatial and temporal editing^35^,^48^, the nature of biological motion – along with its departure from perfect temporal and spatial precision – was preserved in the present stimuli.

The 3-D motion data of each selected cycle were presented as point-light display on a 2-D monitor, using routines of Psychophysics Toolbox version 3^51^ running on Matlab® R2012b (Mathworks). The function *moglDrawDots3D* allowed for depth perception in a 2-D display. The PLF was represented by 13 white discs against a black background, each of which subtended 0.4 degrees of visual angle (°). The whole PLF subtended approximately 5° and 12° when viewed at 80 cm. The PLF was displayed facing the observers, in a configuration as if the observers were watching from 20° to the left of the PLF, which served to optimize depth perception of biological motion in a 2-D environment.

#### Procedure and design

The experimental program was controlled by a customized Matlab script using Psychophysics Toolbox version 3 routines running on a Mac OSX environment. The visual stimuli were displayed on a 17-inch CRT monitor (Fujitsu X178 P117A) with a frame frequency of 100 Hz at a spatial resolution of 1024 × 768 pixels. Participants sat with a viewing distance of 80 cm. The trajectories of finger taps were recorded as positional data by an ultrasonic 3-D motion capture system, Zebris (Zebris Medical GmbH, Isny, Germany), at a sampling rate of 200 Hz. A sensor was attached to the foremost joint of participants’ index finger of the dominant (right) hand during the experiment. Participants tapped at a fixed position marked by a cross on an even, solid surface of the desk, which produced the usual tactile feedback (see^48^ for description of the same setup). The experimental program running in Matlab also triggered the recording of the Zebris system on a trial basis. Participants wore closed studio headphones (AKG K271 MKII) for noise cancellation.

#### Tuning task

Participants self-initiated each trial by pressing the space key. On each trial, a PLF was shown performing either a *Charleston* or a *Balboa* sequence cyclically in one of the six tempi, either with or without TM. Participants’ task was to identify a regular tempo from the movement patterns that they felt was natural to them; the search was not speeded. Once found, they were required to start tapping their index finger regularly in this tempo in a synchronized manner along with the ongoing sequence. Participants were instructed not to switch to a different tempo during a sequence after they started tapping. In total six complete movement cycles were presented on each trial, equaling 48 bounces or 24 limb movements.

#### Spontaneous motor tempo task

To examine whether the tendency of tuning was related to each participant’s preferred tempo, an additional self-paced tapping task was included to measure each person’s spontaneous motor tempo (SMT^52^). Each participant was asked to tap their index finger regularly in a comfortable tempo in the absence of any pacing signal, which was expected to reflect an individual’s preferred tempo. The SMT was measured four times throughout the entire experiment, each lasting 30 s.

The main experiment (tuning task) followed a 2 (Dance style) × 2 (TM) × 6 (Tempo) design, presented in 8 blocks of 24 trials each, with all the conditions balanced across blocks and the order of conditions randomized within a block. Participants underwent six practice trials (and more if needed) before starting the first block. The SMT task was implemented prior to block 1, after block 4, prior to block 5, and after block 8. The entire experiment lasted around 2 hours, completed in two sessions of four blocks each on different days.

### Data analysis

#### Tuning task

The timing of each tap was extracted from the lowest position of each periodic finger trajectory (see^48^ Exp 2). For each trial, all the taps were identified and the inter-tap intervals (ITIs) between successive taps were calculated (in ms), based on which the median of ITIs and the coefficient of variations (CV = SD/mean × 100%) was computed. The median ITI indicated the perceived tempo of each movement sequence. Two criteria were imposed^13^ to determine whether tuning was successful in a given trial: (1) The median ITI did not deviate more than 15% from either the nominal IBI of the bounce (i.e., the metronome IBI) or of the limbs (twice the metronome IBI). (2) The CV did not exceed 15 (except for an occasional omission of one tap as verified by visual inspection of the data). These values were greater than those used in studies employing simple metronomes^53^, as the present biological motion stimuli were more complex and temporally variable in comparison. More variability in the synchronization behavior was thus expected. In the rare occasions that a participant switched tempo after no more than the first three taps in a trial (verified by visual data inspection and calculating the ratio between successive ITIs), if the rest of the taps met the criteria described above, then the identified tempo was calculated from the rest of the taps without the initial, ‘deviant’ ITIs.

Tuning was considered successful in trials that met these criteria, whose data were included in calculating the percentage of tuning per condition. For a given trial, a median ITI within 15% deviation from the metronome IBI indicated tuning to the bounce, whereas that within 15% deviation from twice the metronome IBI indicated tuning to the limb movement. For each participant in each experimental condition, the frequency of trials with tuning to the bounce and trials with tuning to the limbs was calculated separately, indexed as percentages of the total trials in that condition. Each percentage could range from zero to 100.

#### SMT task

The median ITI was calculated for each SMT trial, and the four median ITIs were averaged per participant to index their preferred tempo.

### Experiment 2: Metrical tempo perception in dance movements

#### Participants

Twenty-one volunteers (six male, mean age 25 years, SD = 2.9), who had participated in Experiment 1, returned to take part in this experiment. They were not pre-selected, but rather responded to the recruitment announcement voluntarily. The music and dance training duration ranged from zero to 15 years (all amateurs), with a mean of 6.2 years (SD = 3.9) and 3.7 years (SD = 5.7), respectively. There were more musicians (17 out of 21) than dancers (8 out of 21), amongst whom six had trained in both.

#### Stimuli and materials

The visual stimuli were the same cycles of PLF dance movements in six tempi as used in Experiment 1, but only those with TM. For the *Charleston*, the arm movements were removed by replacing the original arm trajectories with those similar to *Balboa*, with the hands placed upon the hips throughout. This manipulation was meant to avoid introducing the potentially additional metrical periodicity communicated by the arm movements in *Charleston*, as the present task focused on the metrical relation between the bounce and the leg movements. The *Balboa* was the same as before. Both dance movements were either natural as presented in Experiment 1, or manipulated such that the bouncing movement of the trunk was removed. This was implemented on the four trunk markers by eliminating changes of their positional data in the vertical dimension, while leaving the other two dimensions intact.

#### Procedure and design

The stimuli and experimental program were controlled by a customized Matlab script and Psychtoolbox version 3 routines running on a Linux Ubuntu 14.04 LTS system. The monitor setup was the same as before. The finger taps were registered by a customized force transducer that was connected to the Linux computer via a data acquisition device (Measurement Computing®, USB-1608FS). Data were collected at 200 Hz, which was controlled and synchronized on a trial basis by the experimental program in Matlab. Participants wore closed headphones for noise cancellation.

Participants watched on each trial a short sequence of the PLF dancing for two complete cycles, encompassing eight leg movements. They were instructed to watch the figure moving as a whole and to memorize the tempo of the leg movements. Right after each visual presentation they were asked to reproduce their perceived tempo of the leg movements by making four consecutive finger-taps on the force transducer (see^54^ Exp 3). No feedback was given during the experiment. Participants self-initiated each trial by pressing the space key.

This experiment followed a 2 (Dance style) × 2 (Trunk movement: with or without) × 6 (Tempo) design. The trials were presented in 8 blocks of 24 trials each, with all the conditions balanced across blocks and the order of conditions randomized within a block. Participants underwent six practice trials before starting the first block. The experiment lasted around 1 hour, completed in one session.

#### Data Analysis

The timing of each tap was extracted by identifying the time point right before the amplitude of the measured force data exceeded a defined threshold. For each trial, the three ITIs were averaged to yield a mean that represented the perceived IBI of the leg movements. Each mean ITI was then transformed into the percentage of deviation from the nominal IBI of the visual stimuli, i.e., the metronome IBI multiplied by two, as the leg movement was about twice as slow as the metronome. Data from one participant were excluded from further analyses, as the reproduced ITIs deviated on average more than 20% from the nominal IBI, suggesting inattention or incomprehension during the task. The total sample size was thus twenty.

## Additional Information

**How to cite this article**: Su, Y.-H. Visual tuning and metrical perception of realistic point-light dance movements. *Sci. Rep.* **6**, 22774; doi: 10.1038/srep22774 (2016).

## Figures and Tables

**Table 1:** Pearson’s coefficient (*r*) between the individual SMT and the extent of tuning to the limbs relative to tuning to the bounce, computed for each of the experimental conditions.

Movement tempo (nominal IBI in ms)	300 ms	350 ms	400 ms	450 ms	500 ms	550 ms
*Charleston* with TM	0.47	0.49	0.66**	0.56*	0.48	0.60*
*Charleston* no TM	0.46	0.54*	0.54*	0.53*^†^	0.60*	0.57*
*Balboa* with TM	0.33	0.48	0.57*	0.52*^†^	0.68**	0.55*
*Balboa* no TM	0.45	0.43	0.57*	0.40	0.39	0.54*

*denotes significant correlations at the level of uncorrected *p* < 0.05, while **denotes significance at the level of uncorrected *p* < 0.01. The adjustment for false discovery rate (FDR) using linear step-up (LSU) procedure rendered all the significant correlations to be *p* = 0.0605 (for original *p* values < 0.03), except for two cases of *p* = 0.07, denoted by ^†^(for original *p* values between 0.03 and 0.05).
